# Coordinative Management of Soil Resources and Agricultural Farmland Environment for Food Security and Sustainable Development in China

**DOI:** 10.3390/ijerph20043233

**Published:** 2023-02-12

**Authors:** Bo Sun, Yongming Luo, Dianlin Yang, Jingsong Yang, Yuguo Zhao, Jiabao Zhang

**Affiliations:** 1 State Key Laboratory of Soil Science and Sustainable Agriculture, Institute of Soil Science, Chinese Academy of Sciences, Nanjing 210008, China; 2 Key Laboratory of Arable Land Conservation, Ministry of Agriculture and Rural Affairs of the People’s Republic of China, Nanjing 210008, China; 3 Agro-Environmental Protection Institute, Ministry of Agriculture and Rural Affairs of the People’s Republic of China, Tianjin 300191, China

**Keywords:** cultivated land quality, agricultural environment, soil and water resources, agricultural engineering, synergetic development strategy

## Abstract

Major problems in China’s pursuit of sustainable agricultural development include inadequate, low-quality soil and water resources, imbalanced regional allocation and unreasonable utilization of resources. In some regions, overexploitation of soil resources and excessive use of chemicals triggered a web of unforeseen consequences, including insufficient use of agricultural resources, agricultural non-point source pollution and land degradation. In the past decade, China has changed its path of agricultural development from an output-oriented one to a modern, sustainable one with agricultural ecological civilization as its goal. First, the government has formulated and improved its laws and regulations on soil resources and the environment. Second, the government has conducted serious actions to ensure food safety and coordinated management of agricultural resources. Third, the government has planned to establish national agricultural high-tech industry demonstration zones based on regional features to strengthen the connection among the government, agri-businesses, scientific community and the farming community. As the next step, the government should improve the system for ecological and environmental regulation and set up a feasible eco-incentive mechanism. At the same time, the scientific community should strengthen the innovation of bottleneck technologies and the development of whole solutions for sustainable management in ecologically fragile regions. This will enhance the alignment between policy mechanisms and technology modes and effectively promote the sustainable development of agriculture in China.

## 1. Introduction

Agriculture is a social and economic activity of mankind that has the longest, widest and closest connection with nature. Sustainable agriculture belongs to the Sustainable Development Goals (SDGs) launched by the United Nations [1]. The essence of agricultural production is the development and utilization of agricultural resources, a process with great ecological and environmental impacts. Meanwhile, agricultural production is affected and restricted by resources and the environment [1].

**Figure 1:**
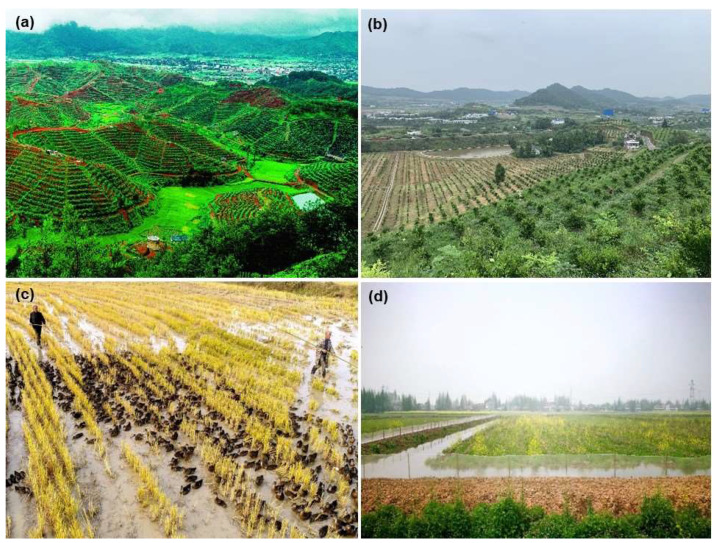
The ecological agriculture models in Southern China: pig raising–biogas production–navel orange (**a**) or citrus (**b**) plantation models in hilly regions, and duck-raising model (**c**) and crayfish culture model (**d**) in paddy fields.

China is faced with huge population, resource and environmental pressures. In its long history of agricultural development, China attaches great importance to relations between agricultural production, resources and the environment. Since the 1960s, China has established ecological agriculture models, such as the pig raising–biogas production–fruit-tree plantation model and multistory cropping-raising patterns in paddy fields and terraced fields (Figure 1) [2,3]. Since the 1980s, China has developed highly intensive agriculture; some inefficient management measures have led to increasingly serious environmental and ecological problems and weaker sustainability [4].

Therefore, sustainable agricultural development has become one of the highest priorities for agricultural policy makers in China. To achieve the Sustainable Development Goals (SDGs), China abides by the principles and patterns of the ecological system when engaging in agricultural production in order to enhance the mutual–complementary relations between agricultural production and environmental protection, optimize the structure and functions of agricultural ecosystems, and constantly improve its overall agricultural production capacity and sustainable development capacity. China has developed an efficient path for agricultural resources and environmental protection by formulating annual and long-term plans for modern agricultural development; constantly enhancing laws and regulations on resources and environmental management; setting up an implementation mechanism for the coordinated management of resources and the environment; and implementing various types of projects for agricultural development in different regions based on coordination between policy systems and technology modes. In this study, we review the keys to success from China’s experience, which could offer models for others engaged in developing sustainable agriculture. First, we address the main problems facing China in sustainably utilizing soil resources and protecting the agricultural environment. Second, the pathway to develop sustainable agriculture and the impact of major national programs since the 1980s are discussed. Finally, we suggest future priorities for both the government and scientific community.

## 2. Methods

First, we undertook a review of the recent literature to evaluate the main problems related to soil resources and the environment for sustainable agricultural development in China. We then undertook a comprehensive review of sustainable agricultural development using four aspects: action plan, regulation system, implementation system and achievement. We used a multi-source review approach that involved assembling information from published scientific and gray literature, including scientific journal papers and reports, official government reports, yearbooks, statistics and webpages. Many of these sources were published in Chinese.

To evaluate the achievements of cultivated land quality improvement projects in China since the 1980s, soil organic carbon density data in the topsoil (0–20 cm) were collected from the National Earth System Science Data Center (http://soil.geodata.cn, accessed on 10 May 2022). The soil pH data in the topsoil (0–20 cm) were collected from the National Cultivated Land Quality Big Data Platform (http://www.farmland.cn/, accessed on 10 May 2022). The soil organic carbon contents and pH values in the topsoil were investigated in the second national soil survey between 1979 and 1984. The Chinese Academy of Sciences launched the “Climate Change: Carbon Budget and Relevant Issues” project and conducted a soil carbon stock survey at a national scale in 2011. The Ministry of Agriculture and Rural Affairs conducted the cultivated land quality survey including soil pH evaluation at a national scale during 2015–2017. SOC stock (tonnes ha^−1^) was calculated by: SOC × BD × Depth × (1 − RF)/10, where SOC is the organic C content (fine soil fraction that passes through a 2-mm sieve, g kg^−1^), BD is the soil bulk density (g cm^−3^), Depth represents topsoil thickness (20 cm in this study), and RF represents the volume fraction of rock fragments (>2 mm). Based on the 1:4,000,000 soil database, the spatial analysis of soil organic carbon stock and soil pH was conducted using the GIS software ArcView 3.2 (ESRI, Redlands, CA, USA).

## 3. Problems with Soil and Water Resources for Sustainable Development in China

In general, the overall agricultural production capacity of China has been constantly improving, with gross grain output ranging from 616 to 669 million/from 2016 to 2020. However, demands for forage grain and industrial grain are increasing rapidly as a result of China’s growing and more urbanized population, optimized dietary mix, and wider use and structural change of agricultural products for industrial purposes. The total grain demand will reach 725 million tonnes by 2030 when considering a population of 1.45 billion and per capita grain demand of 500 kg. Structure-wise, China falls short of the supply of certain agricultural products; the already large gaps may continue to expand in the future. In 2020, China imported 142.62 million tonnes of grain, including 100.33 million tonnes of soybean, 11.3 million tonnes of maize and 8.38 million tonnes of wheat. In addition, the increases in consumption per capita of vegetables, fruits, poultry and milk are estimated to be 5.7%, 13.7%, 14.8% and 44.0%, respectively, from 2020 to 2030. Therefore, a shortage of supply will be normal for China’s agricultural development in the long run [5]. The pressure to ensure food security will lead to the intensive use of soil and water resources.

### 3.1. Shortage of Cultivated Land and Water Resources

China’s cultivated land was 134.87 million hectares in 2019. There is only 0.087 hectares of cultivated land, which represents 36% of the global average (0.24 hectares). In addition, the large majority of arable land has been utilized, and only approximately 5 million hectares of reserved arable land can be developed and utilized on scale in China [6].

China had a total water resource amount of 2904.1 billion m^3^ in 2019, with an annual per capita amount of 2124 m^3^, which was 25% of the world average [7]. On average, agriculture in China faces a water shortage of more than 30 billion m^3^ every year, and some regions are troubled by polluted water resources and shortage of water supply induced by poor infrastructure. In the North China Plain, groundwater overexploitation has led to a decline in the groundwater level in irrigated areas and formed a 4.1-million-hectare funnel-shaped area. In the region with the deepest groundwater level, i.e., in Hengshui city, the deep groundwater level experienced an annual decrease of 1.16 m from 2000 to 2014; however, it experienced an increase of 2.76 m from 2015 to 2016 and an increase of 0.26 m from 2019 to 2020 as a result of the exploitation–supplement balance strategy.

### 3.2. Low Quality of Soil Resources

In 2019, low-quality cultivated land—i.e., levels 7–10, as cultivated land is classified into 10 levels in China, with level 1 being the best and level 10 being the worst—and mid-quality cultivated land (levels 4–6) accounted for 31.2% and 46.8%, respectively, of China’s national total [8]. Overall, the contribution rate of basic land productivity to the total grain yield (wheat, corn and single-season rice) is 45.7% to 60.2% [9], and there is a yield gap ranging from 20% to 40% [10,11]. If measures are taken and a piece of mid-quality cultivated land is upgraded by 1 level, there will be an increase of at least 80 billion kilograms of total grain production. The overall water quality of surface water was good in 2020. Among the 1940 state-controlled surface water monitoring sections, 12.6% were Grade IV and above (surface water is classified into Grade I, II, III, IV, V and above V in China, with Grade I being the best) [12].

### 3.3. Uncoordinated Distribution of Soil and Water Resources

First, there is an uncoordinated spatial distribution of natural factors such as light, temperature, soil and water. Regionally, with a humid climate and abundant water resources, eastern China (47.6% of the national total area) has 90% of the country’s cultivated land, while arid, semi-arid or Alpine western China (52.4% of the national total area) has a mere 10% of the country’s cultivated land. In addition, there is a mismatch between water resources and soil resources. Southern China has more than 80% of the country’s water resources and less than 40% of the cultivated land, while northern China has 20% of the water resources and 60% of the cultivated land [13,14].

Second, there is a mismatch between agricultural production layout and natural resource distribution. In recent decades, the national grain production center has gradually shifted from the southern region with abundant water and heat resources to the northern region facing severe water shortages [15]. At present, with only 15% of China’s water resources, Northeast China and the Huang-Huai-Hai region contribute 53% of the national total grain output. The export of commodity grain to the southern region caused a deficit in water resources. In 2012, the total grain transportation amounted to 79.45 million tonnes from northern to southern China, which equals a virtual water flow of 82.6 billiom m^3^ [16]. However, the “South-to-North Water Diversion Project” transported approximately 6.43 billion m^3^ of water annually from 2014 to 2020. Thus, the resource gap cannot be closed.

### 3.4. Inefficient Utilization of Soil and Water Resources

According to FAO statistics, with only 8% of cultivated land in the world, China feeds 19% of the global population. China’s cultivated land utilization intensity is high: 2.2 times that of the U.S. and 3.3 times that of India. While the U.S. has a well-established fallow system covering a large amount of its cultivated land, China is taking intensive measures such as increasing the multiple-cropping index to ensure that we produce more with fewer land resources. As a result, China’s high agricultural output comes at the price of a low resource utilization rate and low labor productivity. At the same time, agricultural infrastructure in China remains weak and is not fully capable of withstanding natural disasters. The effective irrigation area accounts for only 51.8% of the total, and the average water productivity is approximately 1.0 kg/m^3^, which is much lower than the developed country figure (1.2~1.5 kg/m^3^) [17].

## 4. Problems of Habitat Soil and Environment Safety for Sustainable Development in China

At present, China’s agricultural resources and environment are affected by both exogenous and endogenous pollution, which aggravates the pollution of soil and water resources and increases risks to agricultural product quality and security. On the one hand, industrial, mining and domestic pollutants are released into the agricultural system, leading to poorer environmental quality and worse pollution problems. On the other hand, the overuse of chemicals (such as fertilizers and pesticides) in agricultural production as well as the inappropriate disposal of agricultural wastes (such as livestock/poultry manure, crop straw and plastic film residues) result in severe non-point source pollution. 

### 4.1. High Fertilizer Consumption and Low Utilization Rate

In 2019, China used 54.04 million tonnes of chemical fertilizers, which was 303.2 kg ha^−1^ if taking into account the agricultural planting area (including orchards) [18]. In recent years, China has taken measures in regard to high fertilizer consumption, including an optimized fertilizer mix based on soil tests for main grain crops. Furthermore, the average nitrogen consumption of wheat, rice and corn is 210, 210, and 220 kg ha^−1^, respectively, which is already below 225 kg ha^−1^, the upper limit set by developed countries to avoid water pollution. However, the average chemical fertilizer consumptions in orchards and protected vegetable fields (including greenhouses, tunnels and padding) were 555 kg ha^−1^ and 365 kg ha^−1^, far more than the safety ceiling [19].

The high fertilizer consumption is accompanied by a low utilization rate. The average seasonal utilization rates of nitrogen, phosphorus and potash in wheat, corn and rice in China were 33% (30~35%), 24% (15~25%), 42% (35~60%), and 10~20%, respectively [20,21]. With the implementation of Zero Growth Action in the use of chemical fertilizers and pesticides since 2015, the average utilization rates of chemical fertilizers increased from 35.2% in 2015 to 39.2% in 2019—and there is still much room for improvement [22].

### 4.2. High Pesticide Consumption and Low Utilization Rate

Green and comprehensive measures for pest prevention/control are not widely used in China. The total consumption of pesticides grew from 0.765 million tonnes in 1991 to 1.808 million tonnes in 2014 (physical volume, including active ingredients and auxiliaries; active ingredient amount is 1/7 of the global figure) and the consumption per unit area increased from 5.12 kg ha^−1^ to 11.0 kg ha^−1^, representing 2.5 times the global average [23]. Additionally, the total consumption of pesticides dropped to 1.456 million tonnes in 2019 [22]. China’s average pesticide utilization rate for maize, wheat and rice crop systems was 39.8% in 2019, which is 20~30% lower than developed countries. Problems including soil and water degradation and loss of biodiversity still exist as a result of the residual pesticides that contaminate water via precipitation, surface runoff and infiltration [24,25].

### 4.3. Low Utilization Rate and Recovery Rate of Agricultural Waste

Livestock/poultry raising in China produces approximately 3.8 billion tonnes of animal waste every year, emitting an amount of nitrogen and phosphorus larger than fertilizer consumption does. Livestock/poultry COD emissions account for more than 90% of China’s agricultural non-point source pollution COD. According to the first national pollution source census in 2007, livestock and poultry farms produced 243 million tonnes of organic waste and 163 million tonnes of urine. Furthermore, the total nitrogen and phosphorus discharge from animal excretion reached 1,024,800 and 160,400 tonnes, respectively [26] (The census includes 1,963,624 discharge sources from medium- to large-intensive units and do not include discharges from small producers). Overall, with the increased consumption of fertilizers and the growing amount of animal waste, nitrogen and phosphorus enter the water through surface runoff, leaching and volatilization, which leads to agricultural non-point source pollution [27].

There were 718.8 million tonnes of straw produced from main crops in 2015 [28], of which 43.2% was used for fertilizer. China consumed 2.41 million tonnes of agricultural plastic films in 2019. The mulch films amounted to 1.38 million tonnes and covered a total area of 17.6 million ha [29]. The widely used ultra-thin plastic film (<0.008 mm) is easily aged, fragile, hard to recover and has a recovery rate of less than 60%. As a result, approximately 580,000 tonnes of film residues are left in the soil every year, causing “white pollution,” impacting soil structure and permeability and eventually hindering crops from utilizing water and nutrients in the soil.

### 4.4. Regional Land Degradation and Soil Pollution

In the past 30 years, China’s cultivated land soil fertility has been enhanced in general. According to long-term monitoring from 1987 to 2015, with conventional fertilization management, the amounts of organic matter and nutrient contents in farmland soil showed an upward trend. The average contents of soil organic matter, total nitrogen, available phosphorus and available potassium were approximately 24.7 g/kg, 1.45 g/kg, 27.7 mg/kg and 133 mg/kg—approximately 13.8%, 2.8%, 123.4% and 46.2%, respectively [30,31].

However, more than 40% of the cultivated land was degraded with intensive agricultural land use [8,32]. Approximately 24 million hectares of cultivated land suffered from soil erosion, 7.6 million hectares suffered from salinization, 17.4 million hectares suffered from acidification, and 2.56 million hectares suffered from desertification. In addition, there was approximately 2 million hectares of cultivated land and 3.3 million hectares of protected vegetable land suffering from continuous cropping obstacles.

Soil pollution in China is mainly caused by heavy metal and pesticide pollution and the unreasonable use of untreated organic manure and mulch films. According to a national survey conducted in 2014, 16.1% of investigated sites were polluted. The main pollutants were cadmium, nickel, copper, arsenic, mercury, lead, DDT and polycyclic aromatic hydrocarbons [33,34].

## 5. Achievements of China’s Sustainable Agricultural Development

### 5.1. Formulating Sustainable Agricultural Development Plans and Goals

In 2015, the Ministry of Agriculture and Rural Affairs (MARA) issued the National Sustainable Agricultural Development Plan (2015–2030), which proposed five key tasks to promote sustainable agricultural development: (a) to optimize the development layout and enhance agricultural production capacity; (b) to protect cultivated land and facilitate sustainable utilization of farmland; (c) to save water resources, enhance the water utilization rate and guarantee agricultural water safety; (d) to control environmental pollution and improve agricultural and rural environments; and (e) to restore agricultural ecology and improve ecological functions [35]. The nation is then divided into optimized, moderate and protected development areas, where different management measures will be taken to optimize the agricultural production layout, focus on main varieties and dominant producing areas, and implement precision management based on permanent basic farmland.

In terms of cultivated land protection, 120 million hectares of cultivated land were preserved to guarantee grain production, in which 53.3 million hectares of high-standard cultivated land were constructed by 2020.

In terms of coordinated management of agricultural resources and the environment, water-saving agriculture was developed to ensure an irrigation water consumption of 372 billion m^3^ and an effective irrigation water utilization coefficient of 0.55 by 2020. The “optimized fertilizer mix based on soil test” approach and green prevention/control technology over pests were used to reach coverage rates of 90% and 30%, respectively. The utilization rate of chemical fertilizers and pesticides reached 40%. In total, 75% of the large-scale livestock/poultry farms were supported by waste treatment facilities, the crop straw comprehensive utilization rate reached more than 85%, and the recycling rate of the agricultural plastic films reached more than 80%.

In 2016, the State Council issued the Soil Pollution Prevention and Control Action Plan, which proposed the following actions: (a) identify the soil pollution status of agricultural land through a detailed survey in 2018 and prioritize the protection of unpolluted and slightly polluted cultivated land; (b) take measures including agronomic regulation and alternative planting to guarantee appropriate parameters of agricultural products before 2020 and safely utilize 2.6 million hectares of mildly and moderately polluted cultivated land; (c) identify no-production zones to strictly manage the utilization of severely polluted cultivated land; and (d) restructure the planting mix so that 1.3 million hectares of land will be recovered or rebuilt into forest/grassland [36].

### 5.2. Improving Regulations and Establishing Research and Administration Systems for Agricultural Resource Management and Environmental Protection

China has formulated a number of environmental laws to clarify the requirements for the protection of agricultural resources and the environment. The Environmental Protection Law, the Environmental Pollution Law of Solid Waste and the Prevention and Treatment of Air Pollution Law and Water Pollution Prevention Law have stipulated the requirements for the entry of solid waste, atmosphere and irrigation water into the agricultural environment. In particular, the Agricultural Product Quality Safety Law issued in 2006 sets up specific chapters on agricultural habitat environmental protection. The State Council promulgated the Basic Farmland Protection Regulations and the Livestock and Poultry Scale Aquaculture Pollution Prevention Regulations to facilitate cultivated land protection, comprehensive utilization and non-polluted treatment of animal waste.

In 2015, the Ministry of Agriculture (now called the Ministry of Agriculture and Rural Affairs) set up 37 ministry-level key laboratory systems for discipline clusters, which comprised 42 key comprehensive laboratories, 297 professional (regional) key laboratories and 269 scientific observation stations. At the same time, the MOA improved modern agricultural technology systems for 50 products; each system consists of a national industrial technology research and development center and a number of integrated experimental stations in major agricultural areas. Linked with the research teams in other national research systems, these systems conduct joint research, set up pilot programs, and offer technical training sessions, policy consultation and contingency services on agricultural green development [37].

In terms of resources and environmental protection, the MOA has been establishing an agricultural resources and environmental protection system since the 1980s, which consists of 2 national stations, 33 provincial stations, more than 300 local-level stations and more than 1700 county-level stations, with a total of more than 12,000 practitioners. In 2017, the MOA set up the Cultivated Land Quality Monitoring and Protection Center, which is responsible for setting up the national monitoring network, carrying out cultivated land quality investigation and evaluation, and developing technologies and products for improving cultivated land productivity.

### 5.3. Carrying Out Fertile Soil Projects and Comprehensive Remediation Projects for Heavy- Metal-Contaminated Soil to Improve Soil Quality

**Figure 2:**
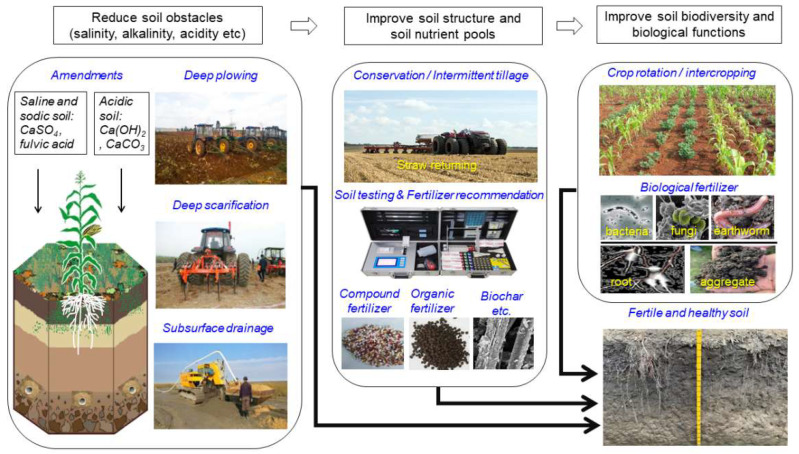
The construction pathway of fertile and healthy soil.

China has perfected the quality monitoring/protection network for cultivated land and improved cultivated land quality. China has established a four-level (national, provincial, city and county), long-term quality monitoring network for cultivated land, which includes 357 national level monitor points and covers 35 major types of cultivated land [31]. Major focuses are middle- and low-yield fields with soil obstacles and soil degradation, such as black soil (Phaeozem) in Northeast China with soil erosion problems, Chao soil (Cambisol) in northern China with groundwater overexploitation problems, and red soil (Acrisol) and paddy soil (Anthrosol) in southern China with soil acidification and heavy metal pollution problems [38,39,40,41]. The construction pathway of high-standard farmland to reduce soil obstacles and improve soil structure, soil nutrient pools and soil biological functions was proposed in different regions (Figure 2) [42,43].

**Figure 3:**
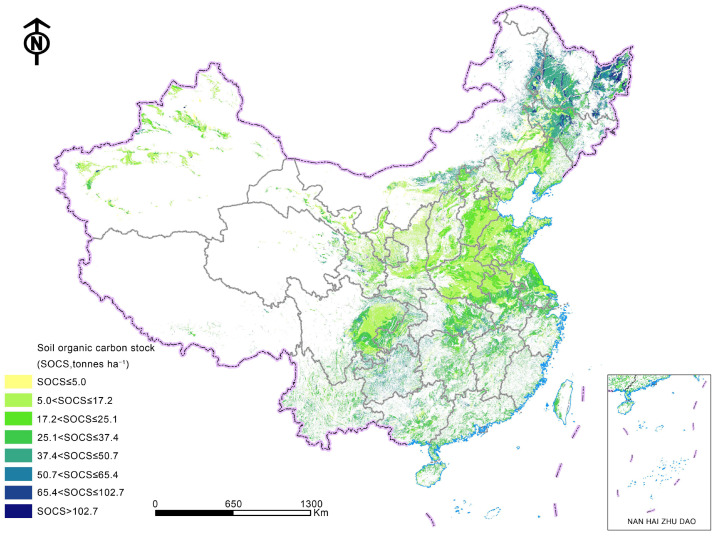
Soil organic carbon stock in the 0–20 cm layer of cultivated land in 1980 in China.

China has implemented a strict cultivated land protection system and comprehensively improved the quality of cultivated land and the ecological environment [44]. Since the 1980s, China has successively implemented the actions of the Fertile Soil Project [45], High-Standard Farmland Construction Project [46], and Northeast Black Soil Conservation Tillage Action [47]. In 2020, organic fertilizers were used in 36.7 million hectares in total, and the planting area of green manure crops was approximately 3.87 million hectares. The average soil organic carbon stock (SOCS) in 0–20 cm of cultivated land was 28.3 tonnes ha^−1^ in China in 1980 (Figure 3), which increased to 33.1 tonnes ha^−1^ in 2011 (Table 1). Only in Northeast China did the average SOCS decrease by 1.05 tonnes ha^−1^. The main reasons for this are the decomposition of soil organic matter with cultivation and soil erosion in rolling hill regions. In other regions, the higher residue inputs following the large-scale implementation of the crop straw return policy increase soil organic carbon density [48]. In general, the average cultivated land quality increased from 4.73 in 2014 to 4.75 in 2019 (a total of 10 levels) [8].

China has carried out investigations on the heavy metal pollution of cultivated land and set up a classified management system. The Ministry of Agriculture released opinions on implementing the *Soil Pollution Prevention and Control Action Plan.* A total of 1.3 million sampling sites have been set up in 108.2 million hectares of cultivated land for dynamic monitoring of heavy-metal-caused soil pollution in agricultural habitats [36]. Integrated physicochemical–biological technology was developed for the remediation of heavy-metal-contaminated soils [49]. Since 2014, the Ministry of Agriculture and Rural Affairs has set up pilot zones for remediating slightly and moderately polluted farmland in 10 provinces. For example, the VIP + n model was adopted in Hunan province, which is represented using rice varieties with low cadmium accumulation (V), adopting reasonable irrigation (I), adjusting the soil pH value (P), and matching assistant measures such as adsorbents and foliar spray inhibitors for reducing the crop uptake of heavy metals (n) [50].

### 5.4. Conducting Water-Saving Agriculture Projects and Zero Growth in Fertilizer/Pesticide Consumption Actions to Improve the Use Efficiency of Resources

China established water-saving agriculture demonstration areas to promote water-saving varieties and technologies, including sprinkler irrigation and water-fertilizer integration [51]. The demonstration program covers more than 26.7 million hectares of farmland. The average effective utilization coefficient of farmland irrigation water in China reached 0.559 in 2020, indicating an increase of 7.7% over 2012 [52].

China has expanded the coverage of the “Soil Testing and Formulated Fertilization Project” from 73.3 million ha in 2010 to 106.7 million hectares in 2016 [53]. Integrated soil-crop system management increased the average yields from 7.2 tonnes ha^−1^ to 8.5 tonnes ha^−1^ for rice; 7.2 tonnes ha^−1^ to 8.9 tonnes ha^−1^ for wheat; and 10.5 tonnes ha^−1^ to 14.2 tonnes ha^−1^ for maize, without any increase in nitrogen fertilizer [54]. By 2020, the coverage rate of green prevention/control for major pest-led diseases reached 41.5% and the use of professional services reached 35.5%. The fertilization rate decreased from 338.3 kg ha^−1^ in 2015 to 303.2 kg ha^−1^ in 2019 (based on agricultural planting area). The total consumption of fertilizers decreased by 10.3% from 2015 to 2019. The consumption of chemical pesticide technical decreased by 19.5% from 2015 to 2019, from 596,500 tonnes to 480,000 tonnes. The utilization rates of chemical fertilizer and pesticide reached 40.2% and 40.6%, respectively, for the three major grain crops of rice, wheat and maize [52].

Since 2017, China has conducted the action for replacing chemical fertilizer with organic fertilizer for fruit, vegetable and tea replacement in 238 counties [55]. The large-scale biogas natural gas pilot project could support a biogas fertilizer production capacity of more than 400 million tonnes every year. China is providing higher subsidies for straw returning, organic manure and thick mulch films (>0.01 mm) and implementing the straw comprehensive utilization project and mulch film recycling project in 143 and 229 counties, respectively. In 2020, the national comprehensive utilization rate of straw and livestock and poultry manure reached 86.72% and 75%, respectively. The residual film processing capacity increased by 186,300 tonnes.

**Figure 4:**
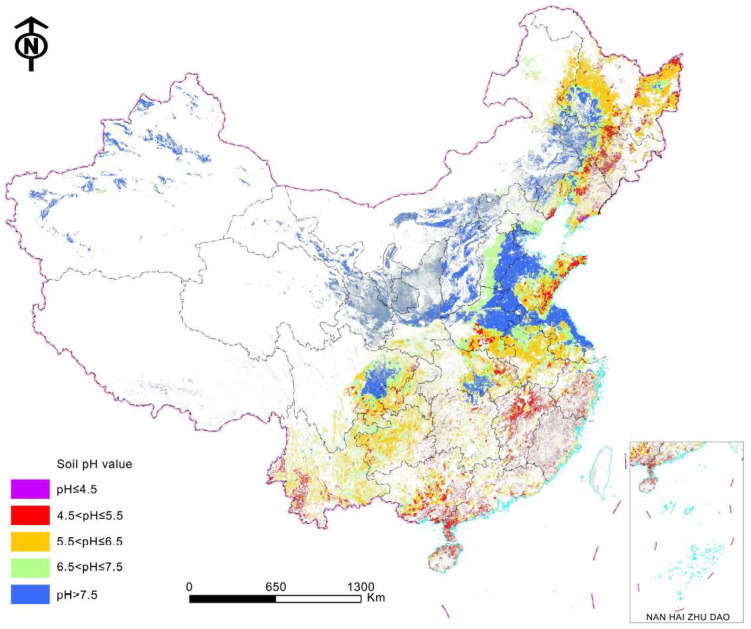
Soil pH in the 0–20 cm layer of cultivated land in the 2010s in China.

However, soil acidification has increased since the 1980s. The average topsoil pH declined significantly by 0.13–0.80 for most soil types from the 1980s to the 2000s, while it remained unchanged for the Aeolian soils with high pH, which accounted for only 9.8% of the total cultivated land in China [56]. For the paddy soils, the average topsoil pH declined by 0.29–0.58 in most major rice-production areas from the 1980s to the 2010s, while it increased by 0.14 in the southeast region (Table 2). The areas of strong (pH < 5.5) and weak (pH from 5.5 to 6.5) acidic paddy soil accounted for 12.9% and 32.5% of the total cultivated land in the 2010s, respectively (Figure 4). The significant soil acidification was mainly caused by the high N surplus, which increased from 142.8 kg ha^−1^ in 2004 to 168.6 kg ha^−1^ in 2015 [57]. Reducing the N surplus while meeting the food demand in 2050 requires an increase in nitrogen use efficiency from approximately 40% to 60% in China [58].

## 6. Future Priorities for the Coordinated Management of Soil Resources and the Agricultural Farmland Environment in China

In recent years, realizing soil health has become a global priority to sustain humans, animals and the environment [59,60]. A large across-the-board increase in soil and environmental quality is required for long-term and sustainable agricultural production in China. While guaranteeing food safety and the supply of major produce, China is paying more attention to the protection of the quantity, quality and ecology of cultivated land through high-standard farmland construction programs. By 2030, 80 million hectares of high-facilitated farmland (62.6% of total national farmland) will be established, with concentrated contiguous land, guaranteed harvests in drought or flood, stable and high yields, and a sound ecology [61]. During this process, a satellite–aviation–ground integrated soil health monitoring network for farmland should be established based on remote sensing, proximal sensing, and prediction models. Moreover, an intelligent decision-making system should be built to propose management strategies for healthy farmland construction.

China has built the Science and Technology Backyard (STB) platform to connect the government, agri-businesses, and scientific community with the farming community, enabling smallholders to sustainably achieve yield and economic gains [10]. China will establish approximately 30 national agricultural high-tech industry demonstration zones in 2025. Each demonstration zone will have a theme that aims to resolve a prominent problem that restricts agricultural green development in China. The main tasks of the demonstration zone include: (a) supporting the entrepreneurship and innovation of new agricultural business entities such as family farms and farmers’ cooperatives; (b) improving innovative service platforms, such as various R&D institutions, testing and testing centers, new rural development research institutes and modern agricultural industry science and technology innovation centers; (c) guiding the scientific and technological resources and talents of colleges and universities and scientific research institutes to gather in the demonstration area; (d) building farmers’ training bases with regional characteristics; (e) improving the ability of information management and service in the whole process of agricultural production; and (f) developing circular ecological agriculture and promoting the efficient utilization of agricultural resources and ecological environment protection.

The government should improve the system for ecological and environmental regulation and set up a feasible eco-incentive mechanism. First, the government should develop a monitoring and evaluation system for sustainable management that covers grain output, produce quality, and eco-resources. Second, the government should compensate and incentivize clean production technology and green production factors that are eco-friendly and resource-saving. Finally, the government should carry out pilot subsidy programs for the use of cycle agriculture technology in ecologically sensitive regions. This will help agricultural producers, agricultural cooperative organizations and eco-professional service systems in performing their role in sustainable management.

**Figure 5:**
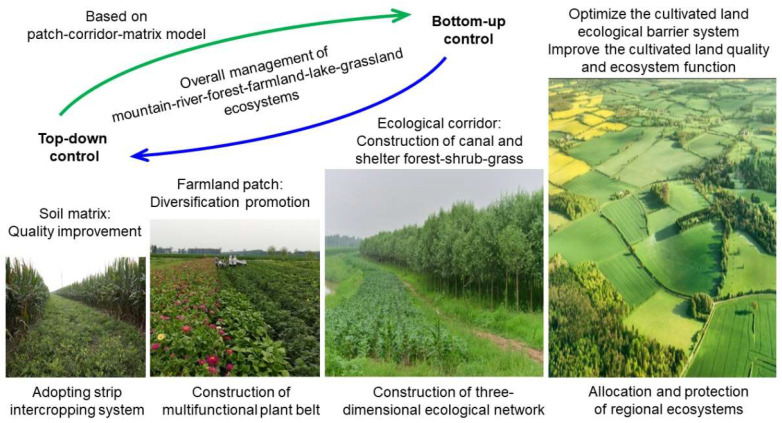
Implement a synergetic development strategy of mountain–river–forest–farmland–lake–grassland–sandy land ecosystems.

For the scientific community, the priority is breaking bottleneck technologies and developing whole solutions for sustainable management in ecologically fragile regions. Ecologically fragile regions such as Northeast China suffer from soil erosion and a decrease in soil organic matter; Southeast China suffers from soil acidification and heavy metal pollution, while Northwest China suffers from soil salinization and climatic aridity. On the one hand, scientists need to develop modern biological technology to solve the bottleneck of nutrient transformation technology in soil-root-microbe interfaces to improve soil quality and nutrient-use efficiency. On the other hand, scientists need to build ecological barriers for controlling and remediating soil degradation and implement a synergetic development strategy for mountain–river–forest–farmland–lake–grassland ecosystems (Figure 5).

In conclusion, the insufficient use of agricultural resources, agricultural non-point source pollution and land degradation are the main problems facing China’s sustainable agriculture. China has improved its laws and regulations and implemented serious actions to ensure food safety and coordinated management of agricultural resources. As a next step, the connections between the government, agri-businesses, scientific community and farming community should be strengthened by building demonstration zones. The government should improve regulation and set up a feasible eco-incentive mechanism, while scientists should break bottleneck technologies and develop solutions for the sustainable management of resources and the environment. This will facilitate sustainable agricultural development in China.

## Figures and Tables

**Table 1:** Changes in the soil organic carbon stock in the 0–20 cm layer of cultivated land from 1980 to 2011 in China.

Vegetation Type	Soil Organic Carbon Stock in 1980 (Tonnes ha^−1^)	Change in SOCS from 1980 to 2011 (Tonnes ha^−1^) **
One crop annually in cold temperate zone	33.2 ± 23.0 *	−1.05 ± 5.75
Two crops annually or three crops for two years and deciduous orchards in warm temperate zone	22.0 ± 16.6	9.75 ± 4.04
Two crops containing upland and rice annually and deciduous and evergreen orchards in transitional subtropics	31.8 ± 15.9	4.64 ± 7.99
One- or double-cropping rice followed by a cool-loving crop or three upland crops annually and evergreen economic crops and orchards in subtropics	28.6 ± 11.9	7.03 ± 4.13
Double-cropping rice annually followed by warm-loving crops and evergreen economic crops and orchards in tropics	29.8 ± 17.1	3.57 ± 5.88

* Data represents the mean value ± standard error. ** Soil organic carbon stock in 2011 was calculated from Table S1 [48].

**Table 2:** Changes in paddy soil pH in the 0–20 cm layer from 1980 to 2010 in China.

Region	Soil pH 1980s	Soil pH 2010s	Soil Acidification Rate from 1980s to 2010s (pH Unit/a)
Northeast China	6.41 ± 0.0074 *	6.07 ± 0.1856	0.0163 ± 0.0006
Middle and lower Yangtze River	5.99 ± 0.0044	5.70 ± 0.0243	0.0006 ± 0.0002
Southwest China	6.15 ± 0.0046	6.29 ± 0.0331	−0.0028 ± 0.0004
Southern China	5.93 ± 0.0040	5.35 ± 0.0243	0.0078 ± 0.0003

* Data represent the mean value ± standard error.

## Data Availability

The soil organic carbon data in the topsoil (0–20 cm) in China are available in the National Earth System Science Data Center (http://soil.geodata.cn, accessed on 10 May 2022) which was built by Institute of Soil Soil Science, Chinese Academy of Science. The soil pH data in the topsoil (0–20 cm) in China are available in the National Cultivated Land Quality Big Data Platform (http://www.farmland.cn/, accessed on 10 May 2022) which was built by Yangzhou Cultivated Land Quality Protection Station, Department of Planting Management, Department of Farmland Construction Management, Ministry of Agriculture and Rural Affairs.

## Supplementary Materials

The following are available online at https://www.mdpi.com/article/10.3390/ijerph20043233/s1, Table S1: Changes in soil organic carbon stocks (SOCS) from 1980 to 2011 for the 58 investigated counties across China.
