# Supplementary material for: Coordinative Management of Soil Resources and Agricultural Farmland Environment for Food Security and Sustainable Development in China
Source: Int J Environ Res Public Health. 2023 Feb 12;20(4):3233. doi: 10.3390/ijerph20043233 (PMC9966783; doi:10.3390/ijerph20043233)
Supplement: Supplementary file 1 [file ijerph-20-03233-s001.zip › ijerph-1793932-supplementary.pdf]

Supplementary Table S1

Table S1. Changes in soil organic carbon stocks (SOCS) from 1980 to 2011 for the 58 investigated counties across China* The table is cited from Zhao, Y.; Wang, M.; Hu, S.; Zhang, X.; Ouyang, Z.; Zhang, G.; Huang, B.; Zhao, S.; Wu, J.; Xie, D.; et al. Economics- and policy-driven organic carbon input enhancement dominates soil organic carbon accumulation in Chinese croplands. Proc. Nat. Acad. Sci. USA 2018, 115, 4045–4050.

County	SOCS in 1980 (Tonnes ha^{-1})			SOCS in 2011 (Tonnes ha^{-1})			Changes in SOCS (Tonnes ha^{-1})		
	Lower	Median	Upper	Lower	Median	Upper	Lower	Median	Upper
One crop annually in cold temperate zone									
Naiman	7.16	8.81	10.47	11.35	13.22	15.12	1.89	4.42	6.92
Wuchuan	22.38	28.13	34.28	24.15	25.93	27.68	−8.47	−2.25	3.82
Baoqing	47.66	54.97	63.70	58.93	66.88	75.00	0.12	11.76	23.07
Hailun	69.46	75.90	82.55	59.74	62.52	65.26	−20.58	−13.40	−6.52
Lindian	39.38	42.80	46.62	37.52	39.00	40.58	−7.91	−3.81	−0.06
Dunhua	67.57	76.94	86.12	65.35	69.60	73.96	−17.37	−7.26	2.93
Gongzhuling	27.46	30.26	32.91	28.51	30.82	33.36	−2.92	0.58	4.26
Yushu	39.48	42.35	45.26	37.88	40.52	43.51	−5.63	−1.79	2.23
Changtu	19.09	20.93	23.02	20.51	21.83	23.20	−1.54	0.90	3.25
Fuxin	16.41	18.18	19.95	18.11	19.40	20.72	−0.95	1.23	3.43
Manasi	18.25	21.52	24.62	19.17	21.52	24.22	−3.95	0.04	4.21
Tabei	16.76	19.42	21.71	14.83	16.56	18.23	−5.75	−2.83	0.27
Ledu	23.21	30.54	39.16	24.04	26.28	28.75	−13.00	−4.27	3.50
Dazi	32.21	36.21	40.22	36.04	38.17	40.47	−2.64	2.01	6.55
Two crops annually or three crops for two years and deciduous orchards in warm temperate zone									
Kenli	8.73	11.31	13.50	14.95	16.93	19.04	2.60	5.66	8.88
Laiyang	11.75	12.89	13.80	19.29	20.58	21.88	6.11	7.72	9.44
Pingyi	11.42	12.28	13.10	20.30	21.97	23.67	7.83	9.70	11.59
Yucheng	9.80	12.58	15.37	25.37	26.89	28.40	11.12	14.30	17.48
Mengcheng	16.60	18.44	20.14	27.10	29.12	31.11	8.03	10.69	13.40
Shuyang	12.91	14.70	16.54	29.58	31.91	34.18	14.25	17.20	20.08
Luancheng	16.19	17.39	18.43	31.62	33.43	35.20	13.96	16.04	18.16
Nanpi	15.33	17.27	19.47	20.86	22.35	23.92	2.43	5.09	7.57
Xiangfen	13.55	18.16	23.50	23.45	26.23	29.05	2.09	8.06	13.51
Yuanping	14.89	18.69	22.99	23.82	28.33	33.42	3.40	9.65	15.93
Ningxian	12.76	13.75	14.79	17.61	18.36	19.07	3.35	4.60	5.84
Zhuanglang	13.80	14.64	15.51	18.79	19.73	20.67	3.81	5.10	6.36
Pingluo	16.12	16.82	17.50	21.69	23.06	24.41	4.69	6.25	7.79
Wugong	16.58	17.47	18.34	27.49	28.38	29.29	9.66	10.92	12.18
Fengqiu	14.98	15.98	17.04	24.57	26.73	29.07	8.32	10.75	13.30
Yuzhou	12.16	15.11	18.62	27.43	29.39	31.36	10.25	14.27	17.82

Two crops containing upland and rice annually and deciduous and evergreen orchards in transitional subtropics									
Dingyuan	11.10	13.26	15.64	25.39	27.57	29.70	11.07	14.29	17.37
Xuancheng	23.31	25.67	28.46	32.60	35.06	37.59	5.61	9.35	12.88
Rugao	14.37	15.98	17.56	21.85	23.10	24.42	5.07	7.13	9.19
Qingpu	37.98	43.02	48.24	37.12	40.07	43.03	−9.10	−2.98	2.86
Tongxiang	32.83	38.26	43.97	25.17	27.32	29.58	−17.10	−10.92	−5.04
Fangcheng	13.91	15.32	16.64	19.64	20.92	22.24	3.77	5.61	7.53
Huangchuan	18.41	19.96	21.51	22.59	24.99	27.55	2.17	5.06	7.98
Xishui	18.80	20.57	22.35	27.71	30.18	32.78	6.51	9.59	12.81
One or double-cropping rice followed by a cool-loving crop or three upland crops annually and evergreen economic crops and ochards in subtropics									
Jinhua	26.41	28.67	31.15	30.47	32.85	35.00	0.73	4.15	7.27
JinXian	26.17	28.94	32.18	38.95	42.29	44.93	8.86	13.26	17.24
Taihe	25.28	30.47	35.75	37.20	39.95	42.80	3.58	9.49	15.34
Jianou	30.91	38.78	46.52	42.10	44.65	47.18	−2.71	5.93	14.58
Guiyang	33.29	36.43	39.76	38.14	42.13	46.20	0.49	5.68	10.75
Wugang	26.50	29.99	33.77	38.93	41.72	44.50	7.11	11.73	16.14
Xingan	30.65	34.70	38.46	34.01	38.26	45.12	−2.43	3.62	11.42
Guanghan	39.12	40.12	41.15	41.07	43.42	45.82	0.72	3.30	5.84
Yanting	13.80	15.48	17.18	26.24	28.05	29.89	10.09	12.59	15.13
Dianjiang	18.35	19.94	21.62	20.75	22.55	24.52	0.11	2.62	5.12
Puding	38.28	41.80	45.37	40.63	44.31	48.25	−2.59	2.53	7.76
Zunyi	28.61	33.24	37.40	32.75	36.23	40.02	−2.49	3.06	8.93
Luliang	29.32	30.39	31.47	40.26	43.80	47.58	9.64	13.41	17.24
Luxi	37.54	41.65	45.57	37.99	41.29	44.83	−5.59	−0.35	5.19
Double-cropping rice annually followed by warm-loving crops and evergreen economic crops and ochards in tropics									
Zhangpu	30.85	36.41	43.29	35.83	38.82	41.62	−5.06	2.32	8.64
Wuming	20.52	22.75	25.00	31.13	35.17	38.70	7.81	12.41	16.71
Gaozhou	19.83	22.43	24.80	29.85	31.83	34.13	6.31	9.42	12.82
Taishan	24.97	29.93	34.45	30.70	33.03	35.37	−2.00	3.17	8.47
Yingde	26.06	29.30	32.43	25.95	28.40	31.01	−4.98	−0.87	3.16
Danzhou	22.35	27.35	32.58	19.17	22.40	25.37	−11.13	−5.01	0.85

*The 58 counties are divided into 5 groups according to crop rotations and climate zones. The “Lower” and “Upper” of soil organic carbon stocks refer to the 95% confidence intervals of the bootstrap estimates.
